# STING Agonist-Induced Skin Inflammation Is Exacerbated with Prior Systemic Innate Immune Activation

**DOI:** 10.3390/ijms24044128

**Published:** 2023-02-18

**Authors:** Marcelina Pyclik, Justyna Durslewicz, Joanna A. Papinska, Umesh S. Deshmukh, Harini Bagavant

**Affiliations:** 1 Arthritis and Clinical Immunology, Oklahoma Medical Research Foundation, Oklahoma City, OK 73104, USA; 2 Department of Microbiology and Immunology, University of Oklahoma Health Sciences Center, Oklahoma City, OK 73104, USA

**Keywords:** innate immunity, STING, diABZI, Toll-like receptor 3 (TLR3), skin, psoriasis, mouse models

## Abstract

Activation of the Stimulator of Interferon Genes (STING) protein has paradoxical outcomes in skin disease. STING activation exacerbates psoriatic skin disease and delays wound healing in diabetic mice, yet it also facilitates wound healing in normal mice. To address the role of localized STING activation in the skin, mice were injected subcutaneously with a STING agonist, diamidobenzimidazole STING Agonist-1 (diAbZi). The effect of a prior inflammatory stimulus on STING activation was addressed by pre-treating mice intraperitoneally with poly (I:C). The skin at the injection site was evaluated for local inflammation, histopathology, immune cell infiltration, and gene expression. Serum cytokine levels were measured to assess systemic inflammatory responses. Localized diABZI injection induced severe skin inflammation with erythema, scaling, and induration. However, the lesions were self-limiting and resolved within 6 weeks. At the peak of inflammation, the skin showed epidermal thickening, hyperkeratosis, and dermal fibrosis. Neutrophils, CD3 T cells, and F4/80 macrophages were present in the dermis and subcutaneous layers. Gene expression was consistent with increased local interferon and cytokine signaling. Interestingly, the poly (I:C)-pre-treated mice showed higher serum cytokine responses and developed worse inflammation with delayed wound resolution. Our study demonstrates that prior systemic inflammation amplifies STING-mediated inflammatory responses and skin disease.

## 1. Introduction

The canonical cGAS–STING pathway responds to extracellular and cytosolic DNA, resulting in the production of type 1 interferons (IFNs) and pro-inflammatory cytokines [1]. STING activation leads to the phosphorylation of TANK-binding kinase 1, followed by the phosphorylation of interferon regulatory factor 3 (IRF3), which translocates to the nucleus and induces type 1 IFNs. Although type 1 IFNs perform critical immune functions, dysregulated IFN production is associated with auto-inflammatory and autoimmune diseases. The gain of function mutations in *TMEM173*, the gene encoding STING, results in STING-associated vasculopathy with onset in infancy (SAVI) [2,3]. The constitutive STING activation in SAVI induces vascular inflammation affecting multiple organs, including the lungs and skin. SAVI patients develop rashes, which worsen over time, leading to blisters and ulcers on the face, hands, and feet. The STING pathway has also been implicated in other skin disorders. For example, increased STING expression is reported in the skin lesions of patients with psoriasis [4,5]. In mouse models, a psoriasis-like skin lesion induced by the topical application of a TLR7 agonist, imiquimod, is exacerbated by systemic injection of 5, 6-Dimethylxanthenone-4-acetic acid (DMXAA), a small molecule STING agonist [5]. Conversely, a genetic deficiency of STING or topical application of a STING antagonist attenuates psoriasis.

**Figure 1:**
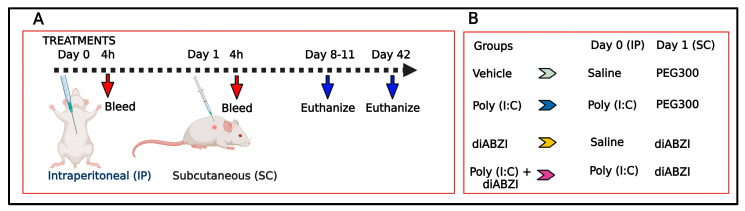
(**A**) Experimental design. Female BALB/cJ mice were given poly (I:C) or saline intraperitoneally on day 0 and diABZI or 40% PEG300 subcutaneously on day 1. Tail bleeds were collected 4 h after each injection for cytokine analysis. The subcutaneous injection site was monitored for skin inflammation for up to 6 weeks. Mice were euthanized at different time points, and skin from the injection site was studied by histopathology, immunostaining, and inflammatory gene expression. (**B**) Four different experimental groups were studied.

STING activation in skin wound healing has paradoxical outcomes. In hyperglycemic mice, STING activation delays wound healing [6], further supporting the pathologic role of STING in skin disease. In contrast, STING signaling (and type 1 IFNs) can facilitate normal wound healing through the increased production of chemokines and immune cell recruitment into the wound [7]. Hence, to investigate the local effects of STING activation in normal skin, we injected mice subcutaneously with diamidobenzimidazole STING Agonist-1 (diABZI). diABZI is a novel synthetic STING agonist with a higher bioavailability and better tissue penetration than natural agonists, making it an attractive candidate for clinical applications [8]. Recently, diABZI-mediated STING activation was shown to be highly effective in controlling SARS-CoV2 infection in experimental model systems [9,10,11,12]. Thus, there is considerable interest in exploring diABZI as a possible anti-viral therapeutic. However, considering that STING exacerbates imiquimod-induced psoriasis [5], we hypothesized that under inflammatory conditions such as a viral infection, STING activation might further amplify pro-inflammatory responses and exert deleterious effects. To test this hypothesis, mice were first injected intraperitoneally with TLR3 agonist poly (I:C) to mimic an acute viral infection. This was followed 24 h later with a subcutaneous injection of diABZI (Figure 1). Our results demonstrate that localized activation of STING in the skin causes a severe inflammatory response, which is exacerbated by prior systemic activation of innate immunity.

## 2. Results

### 2.1. Skin Lesions Induced by STING Activation Are Exacerbated by Prior Systemic Poly (I:C) Treatment

**Figure 2:**
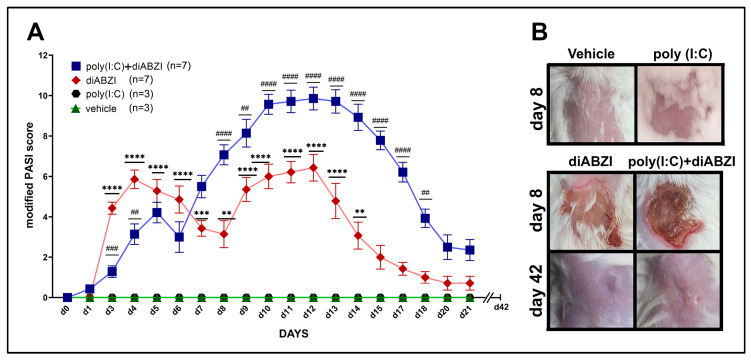
PASI scores at the site of injection. (**A**) Kinetics and severity of diABZI-induced skin inflammation in mice with or without poly (I:C) pre-treatment. BALB/cJ female mice injected with diABZI on a shaved back were evaluated for erythema, scaling, and induration. Each criterion was scored from 0–4, with 0 = no change and 1–4 corresponding with increasing severity. A cumulative modified PASI score was calculated. Data points are mean ± SEM for mice in each group. The number (*n*) of mice in each group is shown in parentheses. Differences between groups were determined using two-way ANOVA followed by Sidak’s post-test for multiple comparisons. Significant differences between diABZI and vehicle controls (*) and between diABZI and poly (I:C)+diABZI (#) are shown., **, ##; *p* < 0.01, ***, ###; *p* < 0.001, ****, ####; *p* < 0.0001. (**B**) Representative images of the lesions on day 8 for vehicle and poly (I:C) control mice and on days 8 and 42 for diABZI and poly (I:C)+diABZI mice.

To investigate the effects of localized STING activation, skin inflammation was evaluated by monitoring the mice daily for erythema, scaling, and induration. The cumulative severity for each group is represented as a modified PASI score (Figure 2A). All the diABZI-injected mice rapidly developed erythema at the site of injection on day 3 (Figure S1A). This was followed by the appearance of skin scaling by day 4 and induration around the area of the lesion by day 9 (Figure S1B,C). The scaling and induration increased in severity and persisted until day 13. The severity of the lesions was significantly higher than in the vehicle-treated control mice.

To investigate the effects of prior systemic innate immune activation on STING-induced inflammation, mice were pre-treated with poly (I:C) through the intraperitoneal route one day before diABZI injection (Figure 1). Compared to those treated with diABZI alone, the mice pre-treated with poly (I:C) showed a lower initial erythematous response on days 4–5 (Figure S1A). However, the redness significantly increased on days 9–14. This corresponded with worse scaling and induration at the site on days 13–15 (Figure S1B,C). The modified PASI scores were significantly higher in the poly (I:C)+diABZI-treated mice compared to the mice treated with diABZI alone (Figure 2A). The mice treated with the vehicle alone or poly (I:C) alone did not develop skin inflammation.

The skin lesions in all the diABZI- and poly (I:C)+diABZI-treated mice underwent spontaneous resolution, and, by day 21, the skin showed minimal changes upon visual inspection. The mice were monitored for an additional 3 weeks, and the experiment was terminated on day 42. At this time, the skin of all the mice appeared completely normal (Figure 2B).

### 2.2. Characteristics of diABZI-Induced Skin Lesions with or without Poly (I:C) Pre-Treatment

The mice were sacrificed at the peak of inflammation (days 8–11), and the skin from the injection site was collected and processed with hematoxylin and eosin staining for histopathologic analysis and with immunofluorescence staining for immune cell infiltrates.

**Figure 3:**
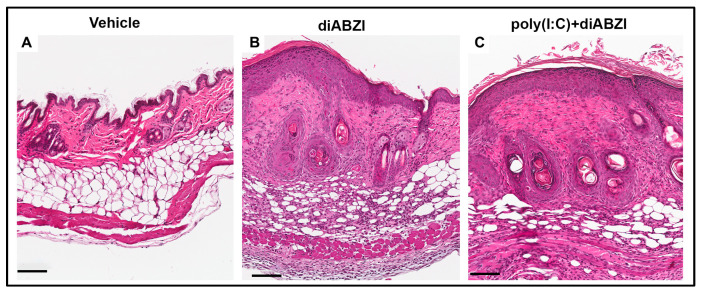
Histopathology of skin lesions at the peak of inflammation. Hematoxylin and eosin-stained photomicrographs of skin collected from the site of injection on day 11, at the peak of inflammation. (**A**) The skin appeared normal in vehicle-treated mice. (**B**) Skin in the margins of the ulcer showed epidermal thickening, dermal inflammation, and fibrosis in mice injected with diABZI and (**C**) poly (I:C)+diABZI (scale bar = 100 µm).

In the diABZI- and poly (I:C)+diABZI-treated mice, the site of scaling and ulceration was associated with significant damage to all skin layers. The ulcerated area showed loss of the epidermis and extensive neutrophilic infiltration (Figure S2). Lymphocytic infiltrates were seen in the deeper layers of the dermis and in the subcutaneous tissues. The skin surrounding the ulcer showed a >3–4-fold increase in epidermal thickness, hyperkeratosis, dermal inflammation, and fibrosis (Figure 3B,C). The lymphocytic infiltration was severe in the deeper dermal layers, extending into the panniculus. Pannicular degeneration was also noted in both the diABZI- and poly (I:C)+diABZI-treated mice.

**Figure 4:**
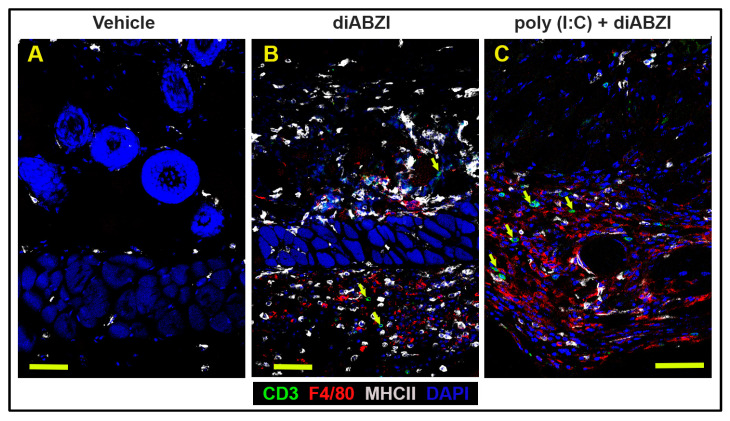
Immune cells infiltration in the dermis and subcutaneous layers at the injection site. Distribution of CD3 T cells, F4/80 macrophages, and MHC II positive cells on day 8 at the site of injection in the skin from (**A**) vehicle-treated, (**B**) diABZI-treated, and (**C**) poly (I:C)+diABZI-treated mice. (**A**) Immunofluorescence staining shows few MHC II expressing cells in the dermis of vehicle-treated mice. (**B**) Deeper layers of the dermis and the subcutaneous tissue show few CD3 T cells (arrows), an increase in F4/80 cells, and MHC II expression after diABZI-,and (**C**) poly (I:C)+diABZI-treatment (scale bar = 50 μm).

Immune cell infiltration was studied at the site of injection (Figure 4). The epidermis and outer dermis in the diABZI- and poly (I:C)+diABZI-treated mice were significantly disrupted compared to those of the vehicle-treated mice. The deeper dermis showed an accumulation of F4/80 macrophages and CD3 T cells (Figure 4B,C). The macrophage and T cell infiltration was associated with the presence of MHC II positive cells. In addition, the inflammatory cells were seen to extend into the subcutaneous space. The vehicle-treated mice showed no dermal or subcutaneous inflammatory cell infiltration (Figure 4A).

**Figure 5:**
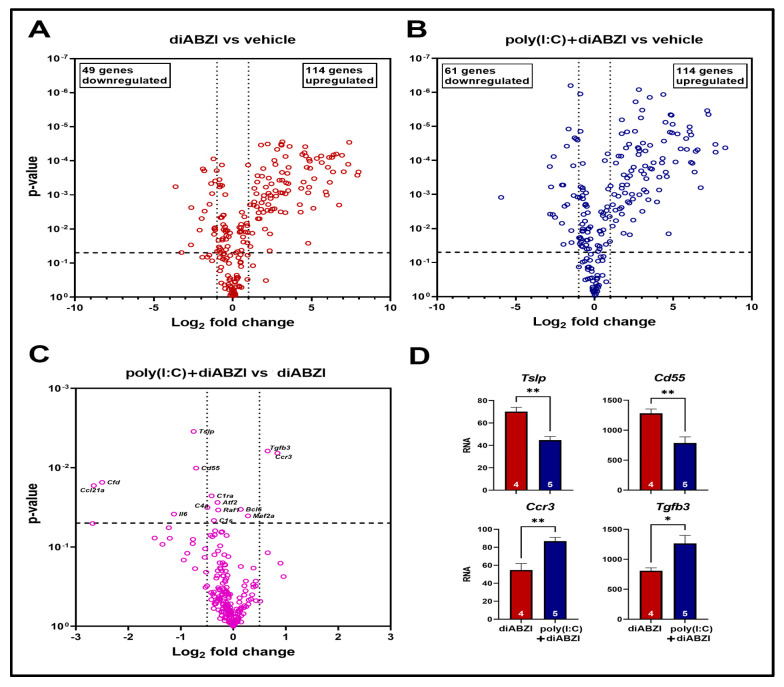
Volcano plots showing differential gene expression from skin harvested on day 8 comparing (**A**) diABZI-treated (*n* = 4) with vehicle-treated (*n* = 3) mice, (**B**) poly (I:C)+diABZI-treated (*n* = 5) with vehicle-treated (*n* = 3), and (**C**) poly (I:C)+diABZI-treated (*n* = 5) with diABZI-treated (*n* = 4) mice. The 13 DE genes are labeled, and the expression values for the 4 most significant DE genes are shown in panel (**D**). * *p* < 0.05; ** *p* < 0.01.

To identify the local inflammatory changes, gene expression analyses of the skin in the early phase of the disease (day 8) were performed using the nCounter mouse inflammation panel, which consists of probes for 254 genes, including 6 housekeeping genes. Compared to the vehicle-treated controls, 163 genes were differentially expressed (DE) in the diABZI group, and 175 genes were DE in the poly (I:C)+diABZI group (Figure 5A,B, Table S1). Of these, 153 genes (109 upregulated, 44 downregulated) were common in both groups.

Further analysis of the DE gene sets showed significant upregulation of the pathways associated with increased cytokine and chemokine activity, interferon and IL17 signaling, and macrophage activation. Although many of the same signaling pathways were upregulated in both the diABZI-treated groups, the top 10 canonical pathways showed higher Z scores in the mice pre-treated with poly (I:C) (Figure S3).

Thirteen genes were found to be significantly different between the diABZI and poly (I:C)+diABZI groups of mice (*p* < 0.05; Figure 5C, Table S1). Poly (I:C) pre-treatment induced the most significant downregulation of *Cd55* and *Tslp* and upregulation of *Ccr3* and *Tgfb3* (Figure 5D). These genes play essential roles in skin inflammation and wound healing. Overall, these data demonstrate that poly (I:C) pre-treatment exacerbates the severity of diABZI-induced skin disease.

### 2.3. Poly (I:C) Pre-Treatment Delays Resolution of diABZI-Induced Skin Disease

**Figure 6:**
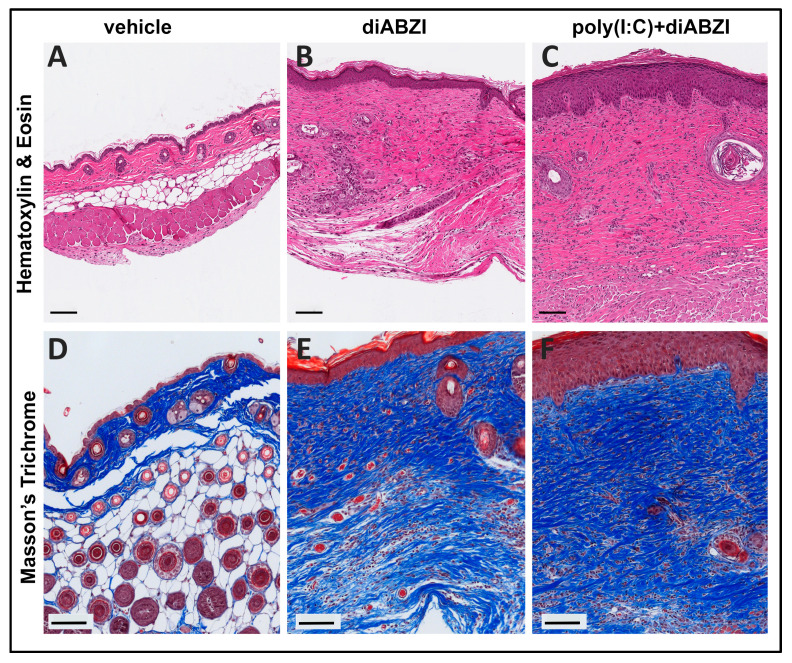
Histopathology of skin lesions in the resolution phase. Representative images of hematoxylin and eosin stained (**A**–**C**) and Masson’s trichrome stained (**D**–**F**) skin from vehicle-treated (**A**,**D**), diABZI-treated (**B**,**E**), and poly (I:C)+diABZI-treated (**C**,**F**) mice on day 42. The poly (I:C)+diABZI-treated mice showed persistent epidermal thickening. In comparison, diABZI-treated mice showed significant lesion resolution with fewer epidermal layers and reduced skin thickness. Extensive collagen deposition indicative of dermal fibrosis was present in both groups (**E**,**F**) (scale bar = 100 µm).

Visually, at 6 weeks post-treatment (day 42), the skin on the back appeared completely normal, with regrowth of hair. The skin surface also appeared normal after depilation (Figure 2B). However, the injection site showed some pathologic changes (Figure 6). Compared to vehicle-treated controls, diABZI- and poly (I:C)+diABZI- treated mice showed increased epidermal thickness, dermal inflammation, fibrosis and some loss of hair follicles (Figure 6A–C). The dermal fibrosis was confirmed by Masson’s trichome staining which showed dense collagen deposition, a concomitant expansion of the dermis, and loss of the pannicular layers (Figure 6E,F). These changes were significantly higher in the poly (I:C)+diABZI-treated mice (severity score 8.0 + 0.77; mean + SEM; *n* = 4) compared to the diABZI-treated mice (severity score 4.6 + 0.66; mean + SEM; *n* = 3, *p* = 0.021). Thus, the poly (I:C) pre-treatment resulted in the persistence of inflammation with a delayed resolution of the skin disease.

### 2.4. Poly (I:C) Pre-Treatment Is Associated with an Elevated Cytokine Response to diABZI

**Figure 7:**
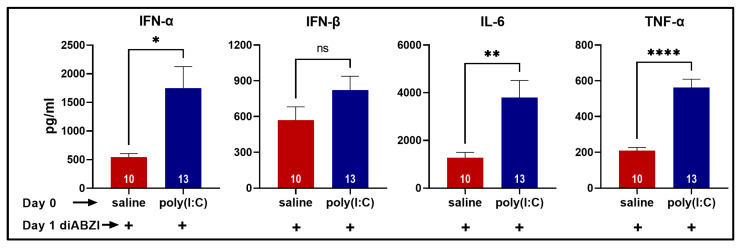
Exaggerated serum cytokine levels after subcutaneous injection of diABZI in mice pre-treated with poly (I:C). On day 0, mice were injected with either poly (I:C) in saline (2 mg/kg) or saline alone, ip. On the following day, all mice were injected with diABZI (2.5 mg/kg s.c.), and tail bleeds were collected 4 h later. Serum cytokines (IFN-α, IFN-β, IL-6, TNF-α) were measured using a multiplex bead-based assay. Cytokine levels in the control groups were below the limit of detection.* *p* < 0.05, ** *p* < 0.01, **** *p* < 0.0001, ns not significant.

Poly (I:C) binds endosomal TLR3 and acts as a potent inducer of type I IFNs and the pro-inflammatory cytokines IL-6 and TNF-α. Serum cytokines were measured in the mice from bleeds collected 4 h after injections on day 0 and on day 1. As expected, on day 0, the poly (I:C)-injected mice had high levels of IFN-α, IFN-β, IL-6, and TNF-α compared to the saline-treated controls (Table S2). Following diABZI injection on day 1, the poly (I:C)-pre-treated mice showed significantly higher serum IFN-α, IL-6, and TNF-α levels than those pre-treated with saline (Figure 7). Notably, cytokines were undetectable in sera collected 4 h after 40% PEG300 injection from mice (*n* = 3) that were pre-treated with poly (I:C). In addition, no cytokines were detected in the vehicle control group (*n* = 3). Thus, intraperitoneal poly (I:C) injection induced a transient elevation in systemic cytokines. However, this temporary systemic inflammation was sufficient to cause a heightened systemic and local response to STING activation.

## 3. Discussion

In this study, we show that a single subcutaneous injection of diABZI, a potent STING agonist, caused a robust local inflammatory response and skin lesions, which persisted for up to 21 days. Interestingly, systemic pre-treatment of mice with poly (I:C) resulted in an exacerbated response to diABZI, with higher systemic cytokine levels, worse skin lesions, and delayed resolution of the skin inflammation. Surprisingly, despite the robust inflammatory response and higher PASI scores, the poly (I:C)-pre-treated mice showed a slight delay in the onset of erythema at the site of the injection. To investigate the cause for this delay, we considered the possibility that poly (I:C) may also induce anti-inflammatory cytokines [13]. Indeed, on day 1, serum IL-10 levels were significantly higher in poly (I:C)+diABZI-treated (102.4 ± 8.9 pg/mL; mean ± SEM) mice compared to diABZI-treated (64.3 ± 10.2 pg/mL; *p* = 0.0183) mice. Thus, while the pro-inflammatory cytokines facilitate disease development, the transient increase in anti-inflammatory cytokines such as IL-10 may contribute to the delayed onset of inflammation.

Although excessive activation of the STING pathway has been linked to autoinflammatory disorders and autoimmunity, STING agonists have also emerged as exciting candidates for anti-cancer and anti-viral therapy [1]. Cyclic dinucleotides such as 2’3’-cGAMP are natural agonists of STING, yet their poor bioavailability and moderate potency have limited their use as therapeutics. Recently, diABZI, a synthetic non-cyclic dinucleotide, has emerged as a novel agonist of STING [8]. It is more potent than 2’3’-cGAMP in activating STING and has demonstrated promising results in tumor regression in a colorectal cancer model [8] and in vitro killing of melanoma cells [14]. In addition, diABZI was also shown to be effective in treating SARS-CoV2 infection in experimental model systems [9,10,11,12]. However, it was reported recently that intra-tracheal diABZI administration induced a rapid neutrophilic infiltration into the lungs and caused acute respiratory distress syndrome [15]. In our model system, diABZI-induced skin inflammation was associated with local neutrophil infiltration, epidermal cell proliferation, hyperkeratosis, macrophage and T cell accumulation, and fibrosis. At the peak of inflammation, the gene expression patterns were indicative of a local cytokine storm, elevated interferon signaling, and macrophage activation. Furthermore, pre-treatment with poly (I:C) amplified the disease. Considered together with the lung report, our study suggests that the beneficial versus pathological effects of diABZI need to be carefully balanced before its application as a therapeutic agent, particularly under inflammatory conditions.

Our study shows that prior activation of innate immunity via the TLR3 pathway significantly influences the subsequent cytokine production induced by STING activation. A single intraperitoneal poly (I:C) injection induced a rapid systemic response indicated by high serum levels of IFN-α, IFN-β, IL-6, and TNF-α. This response was transient, and the cytokines were undetectable after 24 h. However, this short-lasting cytokine spike was sufficient to amplify the response to diABZI. Interestingly, at the peak of the skin inflammation on day 8, only low levels of IL-6 were detected in diABZI-treated (mean ± SEM 59.4 ± 24 pg/mL; *n* = 3) and poly (I:C)+diABZI-treated (mean ± SEM 51 ± 14 pg/mL; *n* = 6; *p* = 0.77) mice. Further, on day 11, all the cytokines were under the detection limits of the assay. These data suggest that the initial amplified cytokine response of the poly (I:C)+diABZI-treated mice makes them more susceptible to exacerbated localized skin lesions and delayed wound healing. To investigate possible mechanisms, we evaluated gene expression at the site of inflammation.

The gene expression analysis on day 8 showed that most genes and pathways activated by diABZI alone or poly (I:C)+diABZI were similar. Thus, the poly (I:C) pre-treatment appears to be an amplification loop for the diABZI response. Of the 13 DE genes between diABZI-treated and poly (I:C)+diABZI-treated mice, the genes showing the most significant differences (*Ccr3* and *Tgfb3* upregulated; *Cd55* and *Tslp* downregulated) are implicated in skin inflammation and wound healing. Skin injury and repair occur in a highly orchestrated environment of chemokines and cytokines [16]. CCR3 is one of the chemokine receptors upregulated within a few days of skin injury before the onset of remodeling and repair. Thus, upregulation of *Ccr3* is consistent with a higher severity of inflammation in the poly (I:C)+diABZI group.

Conversely, reduced local expression of *Cd55* in this group of mice might be associated with delayed wound resolution. CD55, also known as decay-accelerating factor (DAF), is anchored on cells via a glycophospholipid. It can dissociate and prevent the assembly of C3 convertase, thereby inhibiting complement activation [17]. Considering that complement deficiency improves cutaneous wound healing [18], in our model system, lower *Cd55* expression might facilitate complement activation and, thereby, delay wound healing. Thymic stromal lymphopoietin, a pleiotropic cytokine encoded by *Tslp*, has been implicated in the pathogenesis of multiple skin diseases [19]. However, it has also been involved in initiating collagen synthesis [20] in wound healing and wound-induced hair regrowth [21]. Thus, in concert with other genes, significant downregulation of *Tslp* might contribute to a higher severity of and delayed wound resolution in the poly (I:C)-pre-treated mice.

The anatomic and physiologic differences between human skin and mouse skin offer significant challenges in modeling human disease [22]. However, the histopathology of the diABZI-induced skin disease mimics some aspects of a psoriasis-like lesion [23]. A key observation is the significant upregulation of the IL17 pathway (Figure S3) and elevated TNF-α (Table S1) in the skin at the peak of inflammation. The novelty of the present model is that a single injection of poly (I:C) followed by diABZI results in the persistence of inflammatory cells in the dermis for up to 6 weeks, even after the skin surface appears clear on visual inspection. This model is different from the genetic models of skin disease that target specific molecules or the imiquimod-induced psoriasis, which require local application every other day and show a very rapid recovery after cessation of the stimulus [24]. Thus, our study provides a novel model system for investigating wound healing mechanisms.

We used intraperitoneal poly (I:C) treatment to mimic an acute viral infection. Considering that localized STING activation in poly (I:C)-pre-treated mice caused a more severe disease, our study underscores the necessity to re-evaluate the proposed use of STING agonists in anti-viral therapy. However, intra-tumoral STING agonist injections have shown promise in cancer immunotherapy [25], and the local administration of adjuvants is being tested to facilitate anti-tumor immune responses [26]. Our results suggest pre-treatment with TLR3 agonists may improve STING-dependent cancer therapy outcomes.

## 4. Materials and Methods

### 4.1. Mice

All mouse experiments were approved by the Institutional Animal Care and Use Committee and were in accordance with the National Institutes of Health guidelines. Female BALB/cJ mice (9–12 weeks of age) purchased from The Jackson Laboratory (Bar Harbor, ME, USA) were used for all experiments. The mice were housed under specific pathogen-free conditions and had unrestricted access to food (5053 PicoLab Rodent Diet 20) and water.

### 4.2. Treatments

Mice were injected on day 1 with a diABZI STING agonist-1 trihydrochloride (GLPBIO, Montclair, CA, USA) in 40% PEG300 (MilliporeSigma, St. Louis, MO, USA) (2.5 mg/kg, 0.05 mL/mouse, subcutaneous (sc) route) on a shaved back. One day before the diABZI injection, on day 0, some mice were pre-treated with poly (I:C) (HMW, InvivoGen Corp, San Diego, CA, USA) in saline (2 mg/kg body weight, 0.2 ml/mouse, intraperitoneal (ip) route). Mice injected with saline (ip) followed by 40% PEG300 (sc) or poly (I:C) (ip) followed by 40% PEG300 (sc) served as control groups. Tail bleeds were collected from all mice four hours after each injection on days 0 and 1. Sera were stored at -80 °C for cytokine analysis. The experimental design is detailed in Figure 1.

### 4.3. Skin Inflammation

The skin on the back of the mice was monitored daily for inflammatory changes. The severity of the inflammation was evaluated for erythema, scaling or ulceration, and induration progressing to adherence to the underlying structures based on the clinical psoriasis area and severity index (PASI) [27]. The scale for each criterion ranged from 0 to 4 (0, none; 1, slight; 2, moderate; 3, severe; 4, very severe). A modified PASI score was calculated as a cumulative score of the three parameters (erythema + scaling/ulceration + induration/adherence).

The mice were euthanized at different time points, and skin from the injection site was harvested. Pieces of the skin were fixed in 10% buffered formalin for histopathology and in 1% paraformaldehyde-lysine-periodate (PLP) for immunostaining and snap-frozen in liquid nitrogen for RNA extraction and gene expression analyses.

### 4.4. Histopathological Evaluation

Skin pieces were fixed in 10% buffered formalin (Epredia, Kalamazoo, MI, USA) for 48 h, transferred to 70% ethanol, and processed for paraffin embedding. Tissue sections (5-micron thickness) were stained with hematoxylin and eosin using standard methods. Images were scanned on an Aperio CS2 digital pathology scanner (Leica Biosystems, Buffalo Grove, IL, USA). An observer, blinded to experimental details, evaluated skin pathology using criteria previously described by Guiducci et al. [28]. Briefly, the presence or absence of ulceration, epidermal thickening and hyperkeratosis, dermal inflammation, and fibroblast proliferation was evaluated. Pannicular degeneration and reduction in adnexal structures were also determined. Skin sections were stained with Masson’s trichrome to assess collagen deposition and fibrotic changes.

### 4.5. Immunofluorescence Staining

Skin fixed in 1% PLP was floated through 30% sucrose followed by a 1:1 *v*/*v* mixture of 30% sucrose:Tissue-Tek O.C.T. compound (Sakura Finetek, Japan) and processed for cryo-sectioning and immunostaining [29]. Five-micron sections were treated with 0.3% Triton X-100 in PBS, and non-specific binding was blocked with PBS containing 1% BSA and 10% normal horse serum. The sections were incubated overnight at 4 °C with fluorochrome-conjugated antibodies to MHC II (clone M5/114.15.2, AlexaFluor488, BioLegend, San Diego, CA, USA; 1:400 dilution), CD3 (clone 17A2; AlexaFluor594, BioLegend; 1:400 dilution) and F4/80 (clone BM8, AlexaFluor647, eBioscience, San Diego, CA, USA; 1:50 dilution) in 1% BSA in PBS. The nuclei were stained with DAPI, and sections were mounted in Prolong Gold (Invitrogen, Waltham, MA, USA). Images were captured on an LSM710 confocal microscope using Zen software (Carl Zeiss Microscopy LLC, White Plains, NY, USA).

### 4.6. Multiplex Cytokine Assay

Cytokine levels (IFN-α, IFN-β, IL-6, TNF-α) in the blood collected 4 h after injection on day 0 and day 1 were measured using Mouse ProcartaPlex assays (ThermoFisher Scientific, Waltham, MA, USA) on Bio-Plex 200 (BioRad, Hercules, CA, USA), in accordance with the instructions of the manufacturer. Cytokine levels were also measured in blood collected at the time of euthanasia on day 8 and day 11.

### 4.7. Gene Expression Analysis

Snap-frozen skin was ground to a fine powder in liquid nitrogen using a mortar and pestle. RNA was extracted from the powdered tissue using an RNeasy fibrous tissue mini kit (Qiagen, Germantown, MD, USA). Gene expression was studied using the nCounter Mouse Inflammation V2 Panel (NanoString Technologies, Seattle, WA, USA). Differential gene expression was analyzed by using nSolver software (NanoString Technologies). Bioinformatic analyses for the identification of canonical pathways were performed using Ingenuity Pathway Analysis software (Qiagen).

### 4.8. Statistics

Prism 9.0 software (GraphPad, San Deigo, CA, USA) was used for all analyses. A normality test was performed on each dataset to determine Gaussian distribution. Student’s *t*-test was used to determine differences between the two groups. Non-parametric Mann–Whitney test was used for non-Gaussian distributions. Two-way analysis of variance (ANOVA) followed by Sidak’s post-test for multiple comparisons was used to compare modified PASI scores over time. A value of *p* < 0.05 was considered to be statistically significant.

## Data Availability

Not applicable.

## Supplementary Materials

The supporting information can be downloaded at: https://www.mdpi.com/article/10.3390/ijms24044128/s1.
