# Supplementary material for: STING Agonist-Induced Skin Inflammation Is Exacerbated with Prior Systemic Innate Immune Activation
Source: Int J Mol Sci. 2023 Feb 18;24(4):4128. doi: 10.3390/ijms24044128 (PMC9960435; doi:10.3390/ijms24044128)
Supplement: Supplementary file 1 [file ijms-24-04128-s001.zip › Revised Pyclik et al_Supplementary DATA.pdf]

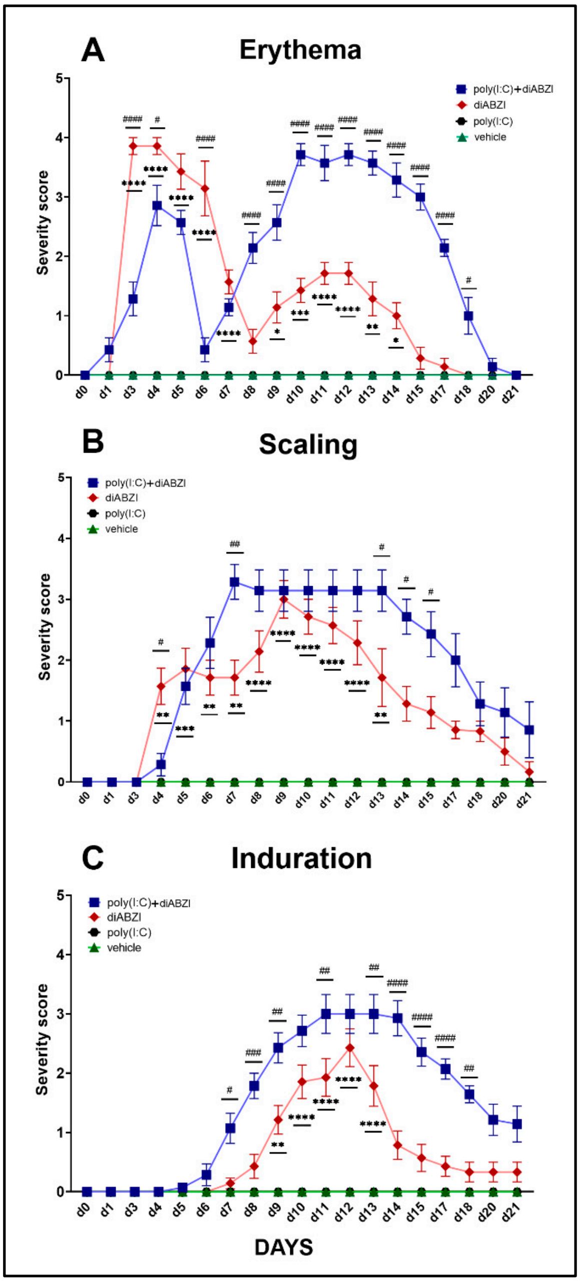

Supplementary Figure S1. Kinetics and severity of individual criteria in the modified PASI score. Quantitative evaluations of the individual clinical scores of **(A)** erythema, **(B)** scaling or ulceration, and **(C)** induration and adherence to the underlying structures were determined on a scale of 0-4, with 0=no change and 1-4 denoting increasing severity. Data points are mean±SEM for mice in each group. Differences between groups were determined using Two-way ANOVA followed by Sidak post-test for multiple comparisons. Significant differences between diABZI and vehicle controls (*) and between diABZI and poly (I:C)+diABZI (#) are shown. *,#; p<0.05, **,##; p<0.01, ***,###; p<0.001, ****,####; p<0.0001

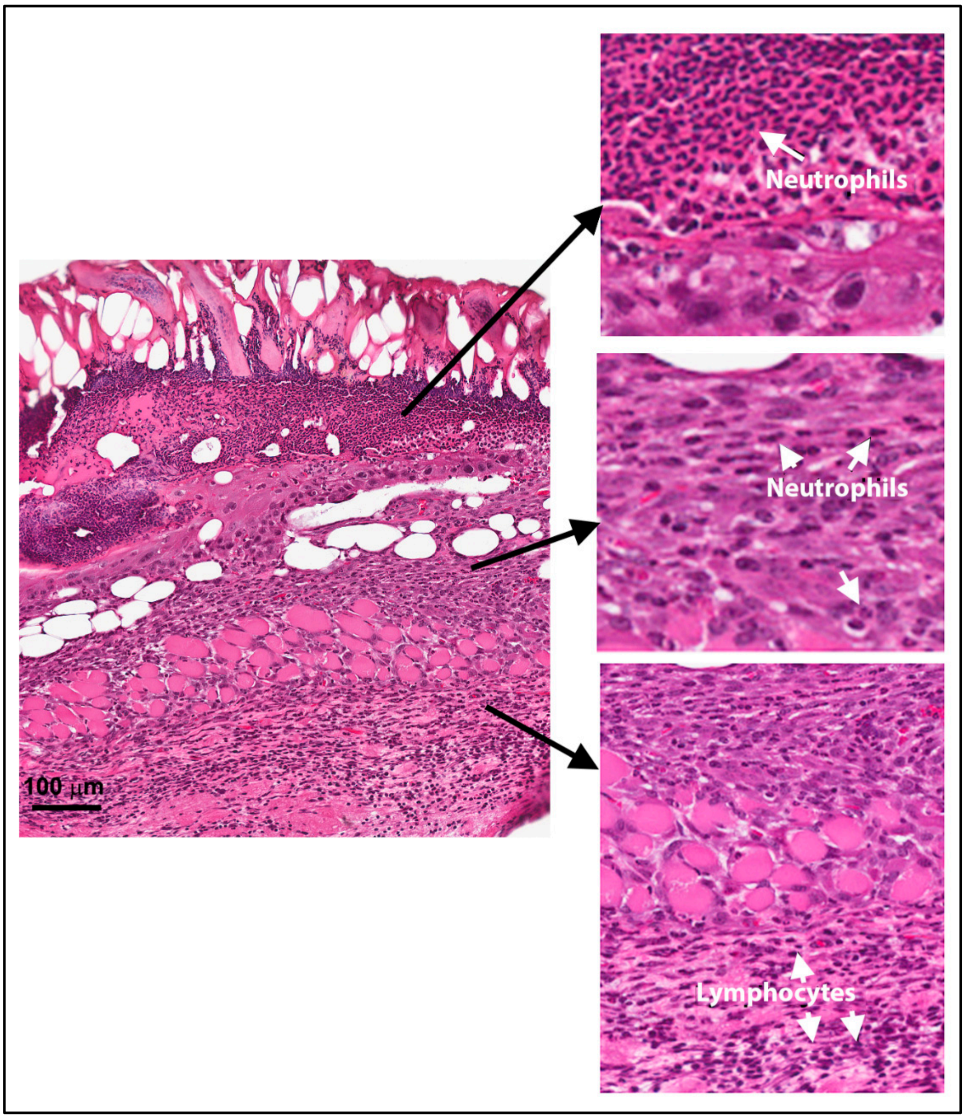

Supplementary Figure 2. Hematoxylin and eosin-stained photomicrographs of skin on day 11, at the peak of inflammation showing neutrophils and lymphocytes at the site of ulceration following diABZI injection.

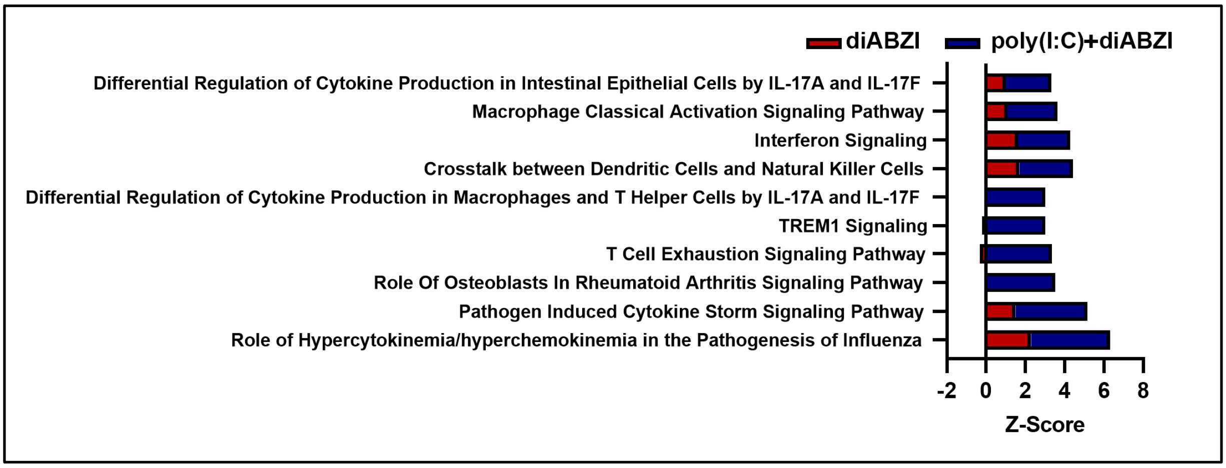

Supplementary Figure 3. Top 10 canonical pathways significantly upregulated in diABZI and poly(I:C)+diABZI treated mice compared to vehicle-treated mice on day 8. The canonical pathways were analyzed using Ingenuity Pathway Analysis software (Qiagen) for DE genes depicted in Figures 5A and 5B.

Supplementary Table 2: Systemic cytokine levels 4h after intra-peritoneal poly (I:C) injection on d0.

**Cytokine**	**Saline** (n=9)	**Poly(I:C)** (n=16)	**p value**
IFN-α	0.00 ± 0.00	117.4 ± 25.58	0.0005
IFN-β	0.72 ± 0.36	77.08 ± 17.98	<0.0001
IL-6	1.37 ± 0.46	270.0 ± 52.46	<0.0001
TNF-α	1.25 ± 0.41	36.82 ± 5.59	<0.0001
